# Analysis of 62 hybrid assembled human Y chromosomes exposes rapid structural changes and high rates of gene conversion

**DOI:** 10.1371/journal.pgen.1006834

**Published:** 2017-08-28

**Authors:** Laurits Skov, The Danish Pan Genome Consortium, Mikkel Heide Schierup

**Affiliations:** 1 Bioinformatics Research Centre, Aarhus University, Aarhus C., Denmark; 2 Department of Bioscience, Aarhus University, Aarhus C., Denmark; Penn State University, UNITED STATES

## Abstract

The human Y-chromosome does not recombine across its male-specific part and is therefore an excellent marker of human migrations. It also plays an important role in male fertility. However, its evolution is difficult to fully understand because of repetitive sequences, inverted repeats and the potentially large role of gene conversion. Here we perform an evolutionary analysis of 62 Y-chromosomes of Danish descent sequenced using a wide range of library insert sizes and high coverage, thus allowing large regions of these chromosomes to be well assembled. These include 17 father-son pairs, which we use to validate variation calling. Using a recent method that can integrate variants based on both mapping and de novo assembly, we genotype 10898 SNVs and 2903 indels (max length of 27241 bp) in our sample and show by father-son concordance and experimental validation that the non-recurrent SNP and indel variation on the Y chromosome tree is called very accurately. This includes variation called in a 0.9 Mb centromeric heterochromatic region, which is by far the most variable in the Y chromosome. Among the variation is also longer sequence-stretches not present in the reference genome but shared with the chimpanzee Y chromosome. We analyzed 2.7 Mb of large inverted repeats (palindromes) for variation patterns among the two palindrome arms and identified 603 mutation and 416 gene conversions events. We find clear evidence for GC-biased gene conversion in the palindromes (and a balancing AT mutation bias), but irrespective of this, also a strong bias towards gene conversion towards the ancestral state, suggesting that palindromic gene conversion may alleviate Muller’s ratchet. Finally, we also find a large number of large-scale gene duplications and deletions in the palindromic regions (at least 24) and find that such events can consist of complex combinations of simultaneous insertions and deletions of long stretches of the Y chromosome.

## Author summary

The Y chromosome is extraordinary in many respects; it is non-recombining along most of its length, it carries many testis-expressed genes that are often found in palindromes and thus in several copies, and it is generally highly repetitive with very few unique genes. Its evolutionary process is not well understood in general because short-read mapping in such complex sequence is difficult. We combine de novo assembly and mapping to investigate evolution in more than 60% of the length of 62 Y chromosomes of Danish descent. We find that Y chromosome evolution is very dynamic even among the set of closely related Y chromosomes in Denmark with many cases of complex duplications and deletions of large regions including whole genes, clear evidence of GC-biased gene conversion in the palindromes and a tendency for gene conversion to revert mutations to their ancestral state.

## Introduction

The human Y chromosome shares around 3 Mb of sequence with the X chromosome—the telomeric pseudoautosomal regions, where recombination occurs. The remainder of the Y chromosome, the male-specific region (MSY) is inherited from father to son without recombination and its evolution therefore reflects mutation, drift, and selection in males and follows a single phylogenetic tree. The MSY consists of interspersed regions of different origins. 1) The X-degenerate regions are directly descended from the chromosome pair that became the sex chromosomes ~180 Mya (million years ago) and have retained 16 genes homologous to X genes, 12 of which are single copy [1], which are thought to be needed for dosage reasons [2,3]. 2) The X-transposed region is unique to humans and originated by a duplication event from the X chromosome (Xq21) approximately 3–4 million years ago [1]. It is now ~99% identical to the homologous region on the X chromosome. 3) The ampliconic regions are all highly repetitive and contain palindromes (inverted repeats) as large as 1.5 Mb with >99.9% similarity between arms. The genes in these regions are thus present in multiple copies and are predominantly expressed in testes [1]. They are thought to affect fertility [4–6], and they potentially also engage in an arms race with genes in similar ampliconic regions on the X chromosome for transmission to the sperm cells during male spermatogenesis [7–9]. Another fascinating feature of the palindromes is that they exchange genetic material between the two arms via non-allelic homologous recombination (NAHR). One effect is that it reduces the divergence between the arms [10,11] and offers a potential way of repairing deleterious mutations, thus escaping Muller’s ratchet. Another effect of NAHR is allowing palindrome arms to exchange material across different palindromes leading to large structural and copy number variants [12].

While these features make the MSY interesting, they also make it difficult to study.

Mapping of short reads to the reference genome (Next generation sequencing or NGS) has been used to survey single nucleotide variants (SNVs), small indels and copy number variants on the Y chromosome [13–15]. However, NGS has limited power when calling variants in highly similar regions such as the ampliconic regions or in the heterochromatic regions, indels that exceed the read length, large chromosomal rearrangements and variants with sequence not present in the reference.

To investigate variation in highly similar regions and potentially find novel sequence on the Y chromosome, *de novo* assemblies show promising results. High-quality assemblies have been constructed for the chimpanzee [16] and the human [1] Y chromosomes, and recently using chromosome sorting and hybrid assemblies also for the gorilla Y chromosome [17]. However, these approaches do not scale well to many individuals from the same species.

The Danish Pan Genome Project [18] provided us with short-read paired-end sequences from libraries with insert sizes ranging from 180 bp to 20 kb for a total of 40X coverage for the Y chromosome. Here we use these data to construct hybrid assemblies for 62 males, including 17 father-son pairs, where we can use concordance of variant calls as a quality measure. From hybrid assemblies and traditional read-mapping we here report a detailed analysis of SNV and structural variation, including standing variation in copy number variants, evolutionary dynamics of the palindromes and estimation of mutation rates.

## Results

### Extracting MSY scaffolds from the hybrid assemblies

We constructed whole-genome hybrid assemblies for the 68 males reported in [18]. We extracted the scaffolds that mapped to the MSY and used them to call variants with respect to the reference genome (GRCh38). We excluded scaffolds that mapped ambiguously to the X and the Y chromosome in the X-transposed region. Six individuals (all fathers) produced poor assemblies, and we excluded these from further analysis (S1 Fig). We therefore based our analysis on the remaining 62 Y chromosomes (27 fathers, one son and 17 father-son pairs).

We were able to recover large scaffolds that on average cover 86.7% of the X-degenerate regions and 41.8% of the ampliconic regions. The reason for the lower coverage of the ampliconic regions is that they mainly consist of palindromes with too high inter-arm similarity to be assembled. In the hybrid assemblies, the palindrome arms are collapsed into one arm with twice the coverage of the scaffolds mapping to the X-degenerate part of the Y chromosome. We show below how they can be partly de-collapsed.

**Fig 1:**
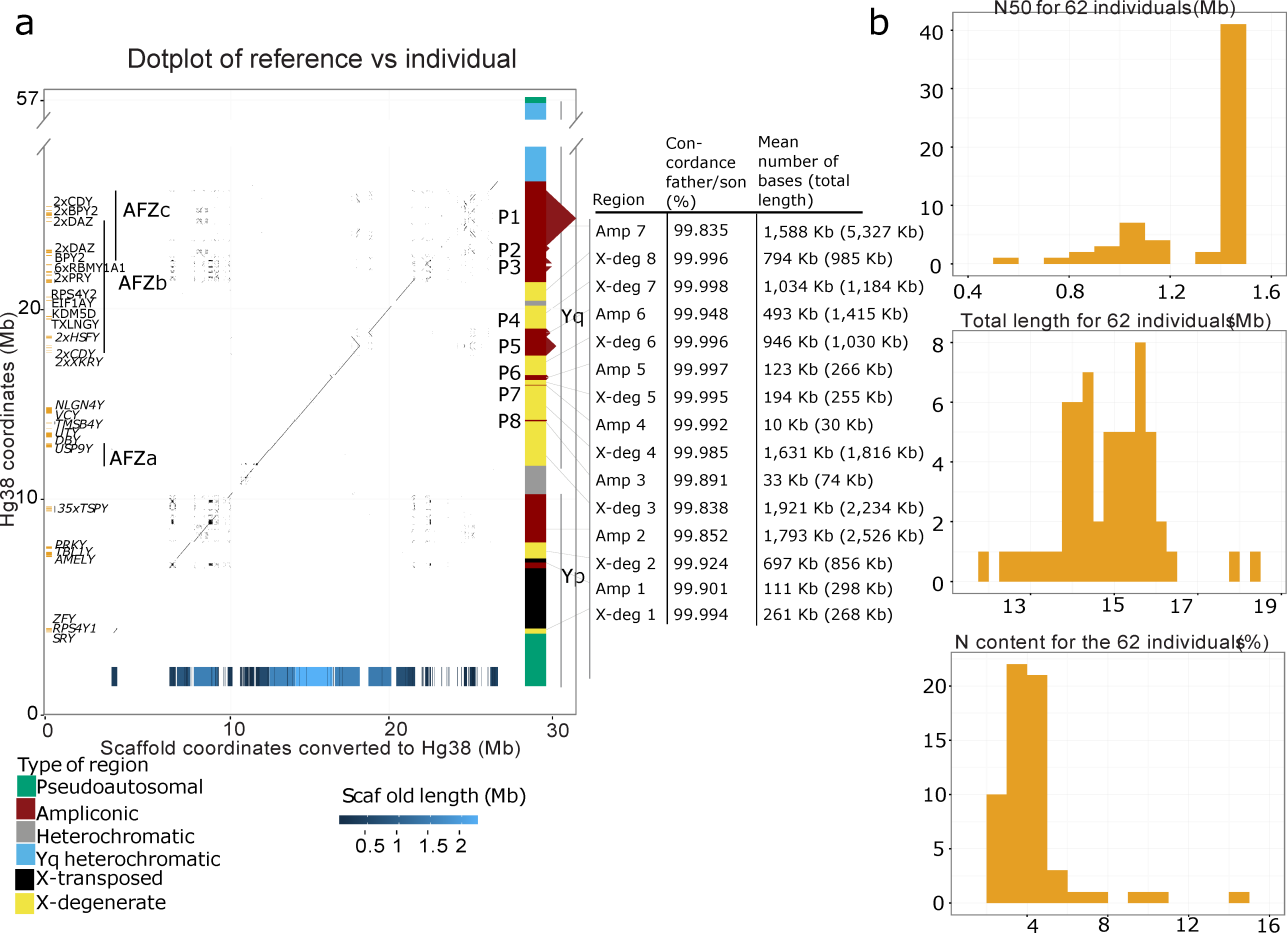
Summary statistics of hybrid assemblies and dot plot of one individual to the reference genome. ***a)*** A dot plot of the reference (GRCh38) vs. one individual (844–01) covering the X-degenerate and ampliconic regions of the Y chromosome. The protein-coding genes and azoospermia factor (AZF) regions are shown on the left, and the different region types and the positions of palindromes are shown on the right. Note that the Y axis has been truncated in the Yq heterochromatic region to show the entire Y chromosome. At the bottom, we show the positions of mapping scaffolds, which are colored according to their length. To the right of the dot plot is a table that shows the average father-son concordance for all sites, the number of sites covered in the region for the 844–01 individual and the total length of the region. **b)** Panels with summary statistics for all 62 individuals with good assemblies. From the top, there is a histogram of the N50 value, a histogram of the total amount of sequence obtained and the gap content of all scaffolds for a given individual.

The scaffolds have an N50 with a mean of 1.29 Mb and median of 1.42 Mb among the individual assemblies. The contigs have a mean and median N50 of 40 Kb. Sequence gaps (patches with Ns) constitute less than 4% and are typically found in blocks (min = 1 bp, max = 20146 bp, mean = 1335.2, bp median = 384.5 bp). Fig 1 shows a dot plot of the scaffolds of one individual to the reference genome. The repetitive nature and collapsed palindromes can be seen as departures from the diagonal.

We evaluated the quality of the scaffolds using alignment between father and son in the seventeen father-son pairs. Differences between fathers and sons can be caused by assembly errors, alignment errors, or *de novo* mutations. To assess the quality of the scaffolds, we excluded repeat masked regions (we excluded 6.9 Mb out of 11 Mb aligned base pairs on average) because they contain low complexity repeats and are therefore prone to alignment errors. For the remaining parts of the scaffolds, any differences between father and son scaffold should mainly be due to assembly errors. The concordance rates between fathers and sons, separated into regions, are also shown in Fig 1. The concordance rates were highest in the X-degenerate regions and lowest in the ampliconic regions but typically, we found between 0.1 and 10 differences in 10,000 bp.

### SNV and large indel calling

We compiled a candidate set of indels and SNVs combining two distinct approaches. First, we called SNVs and indels using traditional mapping approaches (BWA-MEM and GATK haplotype-caller module, henceforth referred to as GATK-HC)[19,20]. Second, we called indels directly from the hybrid assemblies using AsmVar [21], which aligns scaffolds to a reference sequence using LAST [22] and then finds differences. We then genotyped the merged set of candidate variants using BayesTyper [23], which assigns genotype probabilities for each variant, in each individual, based on the k-mer footprint of the variants and the k-mer distribution in the raw reads. We labelled variants already in dbSNP142, 1000 Y [13] or 1000 genomes phase 3 [24] as known and show both the number of variants and the proportion of these that are already known in Table 1. Variants are denoted complex if they cannot be changed into the reference by a single deletion or insertion event. We provide a full list of variants in Supplementary file S2 Dataset.

**Table 1:** Summary of the variants found in this study and their validation rate. We have divided the variants into the heterochromatic region and the combined ampliconic and X-degenerate regions and into whether they occur in more than one haplogroup. The number of variants and the percentage of these that are already known (in parenthesis). The in silico concordance rate is how often an alternative variant found in one member of a family is found in the other member.

	Heterochromatic (0.9 Mb called)		ampliconic and X-degenerate (11.6 Mb called)	
	Recurrent mutations	Non recurrent mutations	Recurrent mutations	Non recurrent mutations
SNPs	7415 (35.7%)	5113 (33.1%)	1730 (31.7%)	5785 (28.0%)
Deletions	295 (21.0%)	204 (17.7%)	569 (17.1%)	651 (20.0%)
Insertions	236 (22.9%)	184 (13.6%)	298 (10.4%)	609 (21.4%)
Complex	0	0	53 (5.6%)	74 (2.7%)
STRs	510 (12.2%)	383 (14.4%)	490 (29.6%)	798 (24.1%)
*In silico* validation rate				
SNPs	0.65	0.99	0.59	0.99
Deletions	0.61	0.99	0.43	0.92
Insertions	0.64	0.99	0.61	0.97
Complex	NA	NA	0.60	0.94
STRs	0.69	0.99	0.69	0.98

We used SNVs in the X-degenerate region (3126 in total) to construct a phylogenetic tree for the Y chromosomes of the 62 individuals using neighbor joining (NJ) and we grouped the individuals based on which mutations they shared. SNV-defined haplotypes (haplogroups) for each family are shown in S5 Dataset. (see S2 Fig; almost identical results were found using maximum likelihood (ML), apart from one individual of haplotype I1a1b1, which did not group with individuals of I1a1b in ML). We divided variants into those occurring once in the phylogeny (non-recurrent) and those occurring multiple times in the phylogeny (recurrent). We report variants for the heterochromatic region separately because the SNV density is much higher here.

A summary of the number of variants is shown in Table 1. We find a very high father-son concordance for non-recurrent variants whereas only about 2/3 of the recurrent variants are concordant among father-son pairs suggesting a rather high false discovery rate for these. There are two primary reasons for this low concordance rate in the recurrent variants. 1) They often occur at positions with multiple similar variants, for instance the reference is C and the alternative alleles are CA,CAAA,CAAAA,CAA. 2) The flanking sequence up and downstream of the variant is very similar to other parts of the genome. Both will not give unique k-mers and therefore the genotyper has difficulties determining the genotype.

We experimentally validated a set of random large indels by Sanger sequencing. We chose to validate these because they had a lower concordance rate than the SNVs. Within the set of non-recurrent variants, 43 out of 43 validated experimentally (29 deletions and 14 insertions) yielding a validation rate of 100%. For recurrent variants, 16 were validated out of 20 yielding a validation rate of 80%. Both are in line with the *in silico* validation rates. Details on the validation results are presented in supplementary file S2 Dataset.

**Fig 2:**
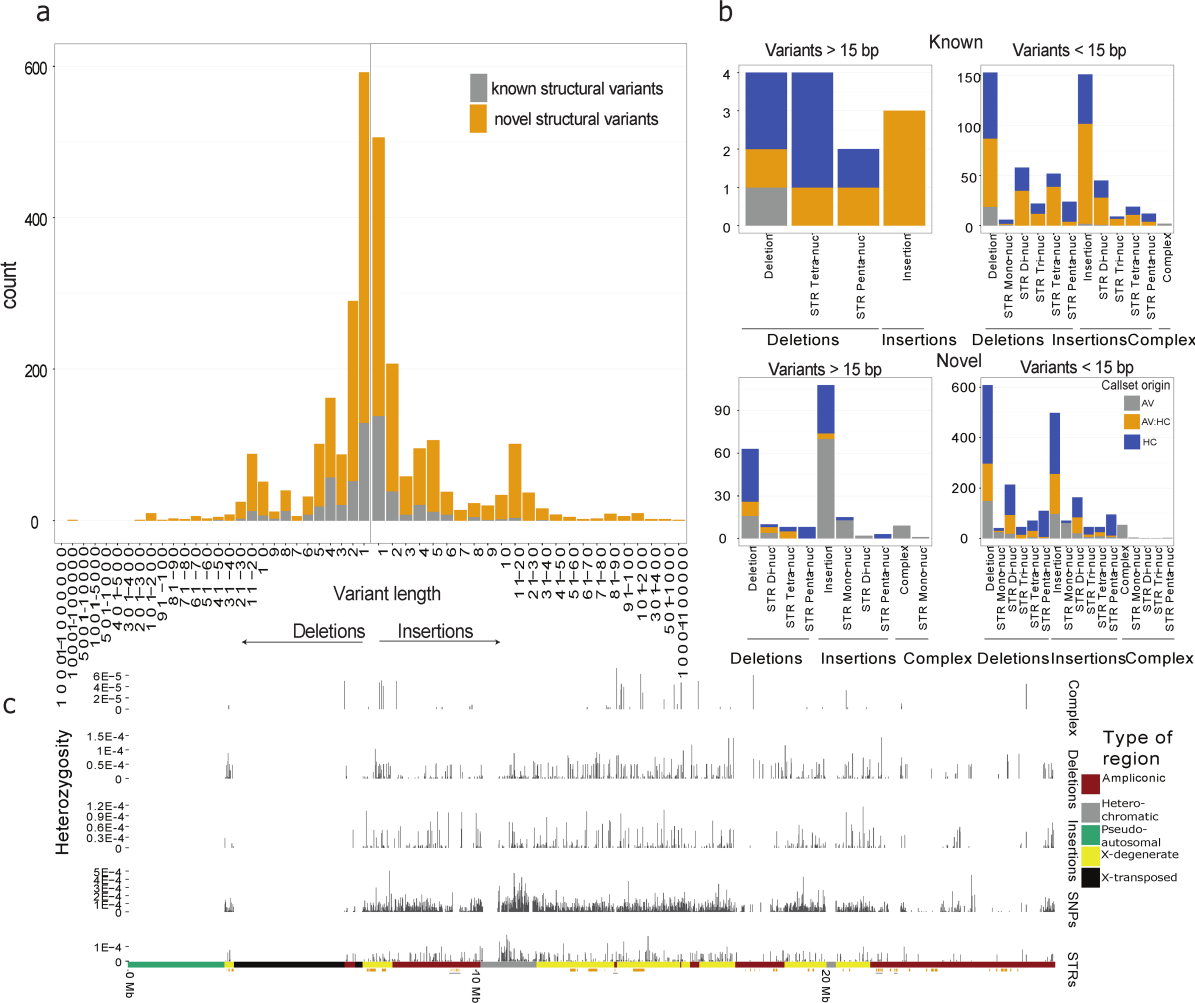
Size distribution, method of discovery and location of large indels. **a)** The size distributions for non-recurrent complex variants, deletions, STRs and insertions. Known indels are colored gray while novel variants are colored orange. **b)** The four bar plots show the known and novel variants grouped into variants larger or smaller than 15 bp respectively, and they are colored according to the method that identified them, which is either GATK-HC (HC) or AsmVar (AV) or both (AV:HC). **c)** The average heterozygosity in windows of 10 kb across the Y chromosome for complex variants, deletion, insertions, SNPs and STRs. We only report regions where more than half the individuals aligned with more than 5000 bp on average and we show the genes of the Y chromosome below.

Fig 2 breaks down the variant set into size classes, call set origin and shows the difference between Y chromosomes along the Y chromosome. The proportion of the variants that have not been observed before (novel) increases with the size of the indels. While the majority of known indels below 15 bp in length are identified by both methods, mapping-based assemblies identifies more short novel variants. The opposite is true for variants larger than 15 bp, especially for insertions where most were identified from the hybrid assemblies.

Even though the Y chromosomes studied here belongs to common European haplogroups (R and I) and haplogroup Q we identify 29 novel variants present in all haplogroup I individuals, 66 novel variants present in all Q individuals and 1 novel variant present in all Rs. We also found 174 Insertions and 104 deletions in all individuals, meaning that this sequence has likely been lost or gained in the reference Y chromosome. We used BLAST [25] to investigate if insertions above 500 bp have similarities to other known sequences. One variant found in all haplogroup I individuals is a 3326 bp insertion that shares 98% identity to a segment on the chimpanzee Y chromosome and thus must have been lost in the lineage leading to the reference Y chromosome that is R1b1.

Because a single phylogeny can be constructed for Y chromosomes, we can estimate how many generations are spanned by the tree using the number of X-degenerate mutations and a X-degenerate specific mutation rate of 3.14E-8, which was estimated based on resequencing of the Y chromosomes of 753 genealogically-connected Icelandic males spanning a total of 47,123 years [26]. We use the number of variants and the length of the callable region to give estimates of the rate at which different variants occur in Table 2. It is apparent that the ampliconic substitution rate is smaller than the rate for the X-degenerate region (discussed further below) and that the estimated rate for the heterochromatic region is much higher than for the rest of the Y chromosome. We note that other calibrations could also be used, e.g. using the rate from [27] of 2.15E-8 (assuming 29 years per generation) would reduce all our estimates by 33% but keep the same relative differences among types of variation and among genomic regions. We report the rate in mutation per position per generation (PPPG).

**Table 2:** The rate of SNPs, deletions, insertions, complex variants and STRs are shown for three classes of region. Note that the rate for the X-degenerate region is the same as [26], because we used it for calibrating the number of generations spanned by the tree.

	X-degenerate (PPPG)	Ampliconic (PPPG)	Heterochromatic (PPPG)
SNPs	3.14E-8	2.76E-8	4.76E-7
Deletions	4.01E-9	2.50E-9	1.90E-8
Insertions	3.73E-9	2.29E-9	1.69E-8
Complex	3.62E-10	3.12E-10	
STRs	4.82E-9	3.69E-9	3.58E-8

Despite the very high estimated substitution rate for the centromeric heterochromatic region, we observe the same high *in silico* validation rate suggesting that this region is indeed extraordinarily polymorphic and not subject to a higher false positive rate.

To investigate possible reasons for the extraordinary polymorphism, we searched for homology of sequence in this region with other parts of the genome. Using BLAT[28] in sliding non-overlapping windows of 50 kb of the centromeric heterochromatic region (10 Mb to 11.7 Mb) we found that 684 kb of the 1.7 Mb had windows of 10 kb with more than 96% similarity to sequence fragments from other chromosomes (see supplementary file S2 Dataset for details). These chromosomes include regions close to the centromere on chromosomes 21, 9, 22, 2 and 16, plus uncharacterized fragments such as Un_GL000218v1. The full list is given in S2 Dataset.

### Understanding palindrome dynamics

**Fig 3:**
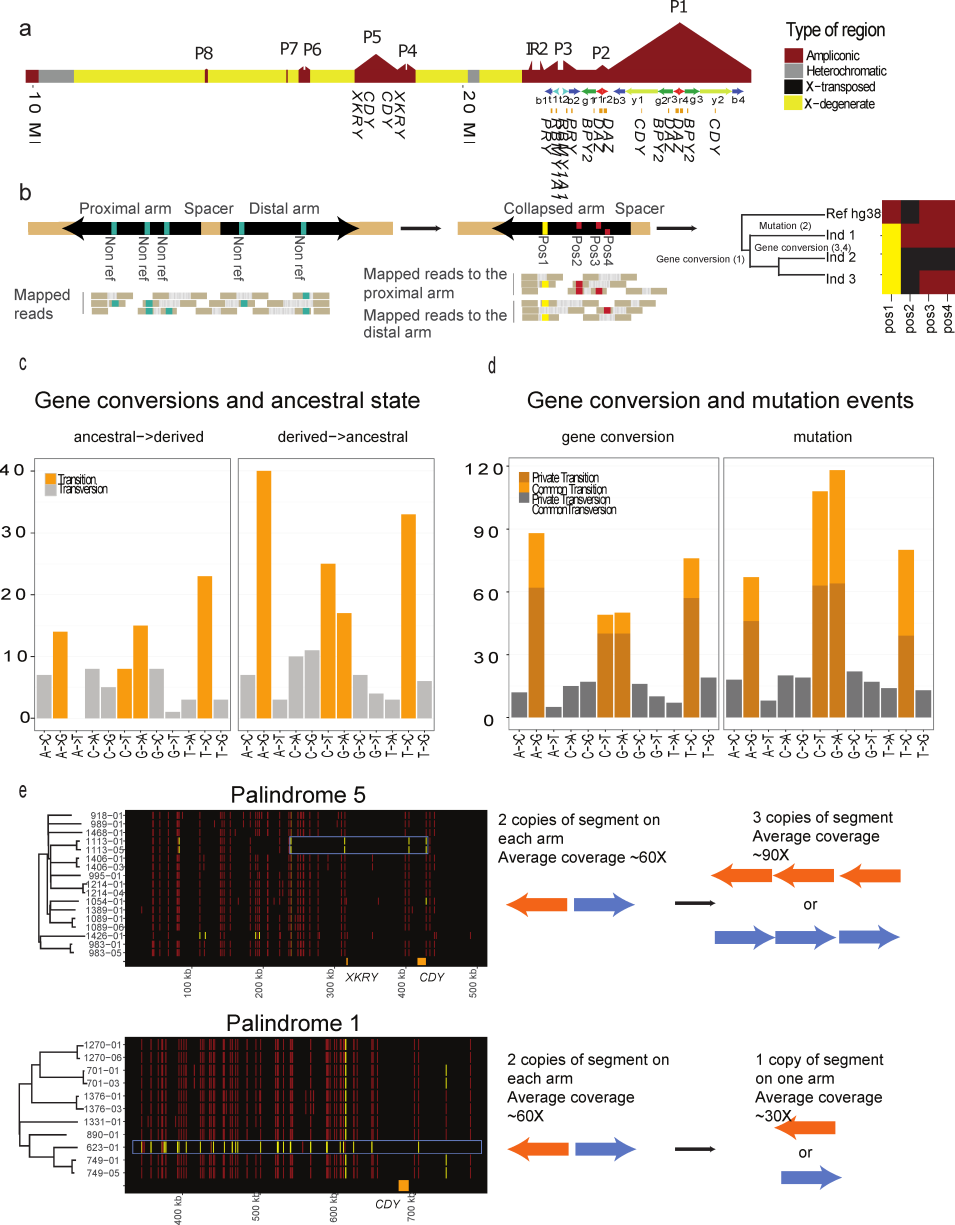
Analysing the dynamics of palindrome arms. ***a)*** An ideogram of the Y chromosome with the different regions colored accordingly to their type. The positions of the palindromes are marked and the protein-coding genes are shown below. **b)** Due to the high similarity of the palindromes many reads will map to both arms and only when there are differences between the arms will the reads map uniquely. However, if only one arm of the palindrome is used the palindrome can be treated as a pseudo-diploid chromosome with differences between arms corresponding to SNVs. Comparing different individuals for which a single phylogenetic tree exists can be used to identify mutations (position 2 in individual 1) and gene conversions (positions 2 and 3 in individual 2 and position 1 in individual 1, 2 and 3). **c)** The gene conversions are grouped based on if the ancestral or derived base is used as a donor sequence. Ancestral->derived means that an ancestral base is converted to the derived base and Derived->ancestral means that the derived based is converted to the ancestral base. The number of gene conversion events for each base transition is shown and the bars are colored according to transitions or transversions classification. **d)** Number of base changes grouped by event type (being gene conversion or mutation). The bars are colored based on if they are transitions or transversions and whether they are found in one individual or father/son pair (private) or in more individuals (common). **e)** Part of the phylogeny and genotypes for palindrome 5 and the y1/y2 segments of palindrome 1 (see part a) are shown. The individuals’ IDs are made up of family ID and a number plus a 01 for fathers and 03 and above for sons. In palindrome 5 the coverage of the segment has increased from around 60X to 90X, suggesting that three copies of this segment now exist. In palindrome 1, the coverage has decreased from 60X to around 30X, suggesting that only one copy of the segment exist.

The two arms of the palindromes are collapsed in the hybrid assemblies due to their high sequence similarity. However, we can investigate palindrome dynamics with respect to mutations and gene conversions using a similar approach to that of another study [10]. The approach is to map reads to one palindrome arm so that differences between arms will appear as pseudo-heterozygous SNV. We mapped reads to all proximal arms of the 8 palindromes and focused on regions with twice the coverage as the X-degenerate region. We therefore removed r1, r2, r3, r4, g1, g2, g3, b1, b2, b3, and b4, because these are present more than twice, retaining 2.7 Mb of sequence (see Fig 3 for naming of segments).

To find mutations and gene conversions, we inferred the ancestral state of each node in the phylogeny based on X-degenerate SNVs. We inferred the ancestral state of the haplogroups present in this study (R,I and Q) using individuals ERR1395549, ERR1347702 and ERR1395593 from the Simons Genome Diversity Project [29], which belong to haplogroups H2b, C2a and E1b, respectively as outgroups. S4 Fig shows the evolutionary relationship between the haplogroups in this study and the haplogroups used as outgroups. We use the estimated split times between haplogroups from [13].

To identify mutation events, we searched for positions where the pseudo-genotype of the parent node was homozygous and the child node was heterozygous. This would mean that a mutation occurred on the branch between the parent and child node, changing one of the bases in one of the palindrome arms making the position look like a pseudo-heterozygous allele. In Fig 3B on the far right, this would correspond to position 2 in individual 1.

To identify gene conversion events, we searched for positions where the pseudo-genotype of the parent node was heterozygous and the child node was homozygous. In Fig 3B, on the far right, this would correspond to positions three and four in the second individual.

We successfully identify 603 mutations and 416 gene conversions in 696 positions across the 7 palindromes. This is a major increase compared to previous studies of 10 positions [10] and 3 positions [11] and allow us to quantify rates and types of mutations and gene conversions. We find that there is a bias towards converting bases into their ancestral state (Fig 3C). We find 100 gene conversions that converted the ancestral to the derived and 171 that converted the derived to the ancestral which is statistically significant (p = 1.61e-05 Chi-square test). In the remaining cases the ancestral genotype was inferred to be heterozygous. We find that gene conversions also show a bias towards GC base pairs with 259 GC conversions and 158 AT conversions which is statistically significant (p = 7.58e-07 Chi-square test). We also have the opportunity to study new mutations within the recent history of the palindromes. We find a mutation bias towards AT base pairs with 336 mutations towards AT bases and 267 mutations towards GC bases (56% versus 44%) which is statistically significant (p = 4.96e-03 Chi-square test). This ratio is similar to what we find when we look at all mutations along the Y chromosome (54% versus 46%). To investigate whether this ratio is affected by gene conversion, we also looked at more recent mutations, private to one individual or one family. We find 186 mutations to AT base pairs and 157 mutations to GC base pairs, which is similar to the rate of all mutations (54% versus 46%), but it is not statistically significant (p = 0.11 Chi-square test) due to the low sample size.

Multiple adjacent gene conversions in an individual can either be explained by many independent gene conversions or a single large gene conversion. The latter would be the most parsimonious. In individual 623–01 we find that almost all pseudoheterozygous positions have been converted into pseudohomozygous positions (see Fig 3E). This suggest that y1 or y2 of palindrome 1 has been deleted and the drop in coverage from ~60X to ~30X supports this finding. In the 1113 family, we find a 150 kb segment where all pseudoheterozygous positions have been converted to pseudohomozygous positions (see Fig 3E). We also find that the coverage has increased in this region from ~60X to ~90X. This suggest that part of one arm has replaced the other arm in a large gene conversion event and then been copied yet again, yielding three identical copies of the segment. This emphasizes that very complex rearrangements of the Y chromosome occur.

To estimate the rate of gene conversions and mutations we used the total length of the palindrome sequence that was analyzed (2.7 Mb) and the generations spanned by the tree found using the X-degenerate SNPs. We find that the gene conversion rate is 1.21E-8 events per position per genome and the mutation rate is 1.76E-8 events per position per generation. The gene conversion rate fits well another study [10] where they estimate it to be between 7.25E-9 events per position per generation and 2.10E-8 events per position per generation. The mutation rate is, however, lower than what was found in another study, which was 2.86E-8 events per position per generation [26].

### Copy number of genes

**Fig 4:**
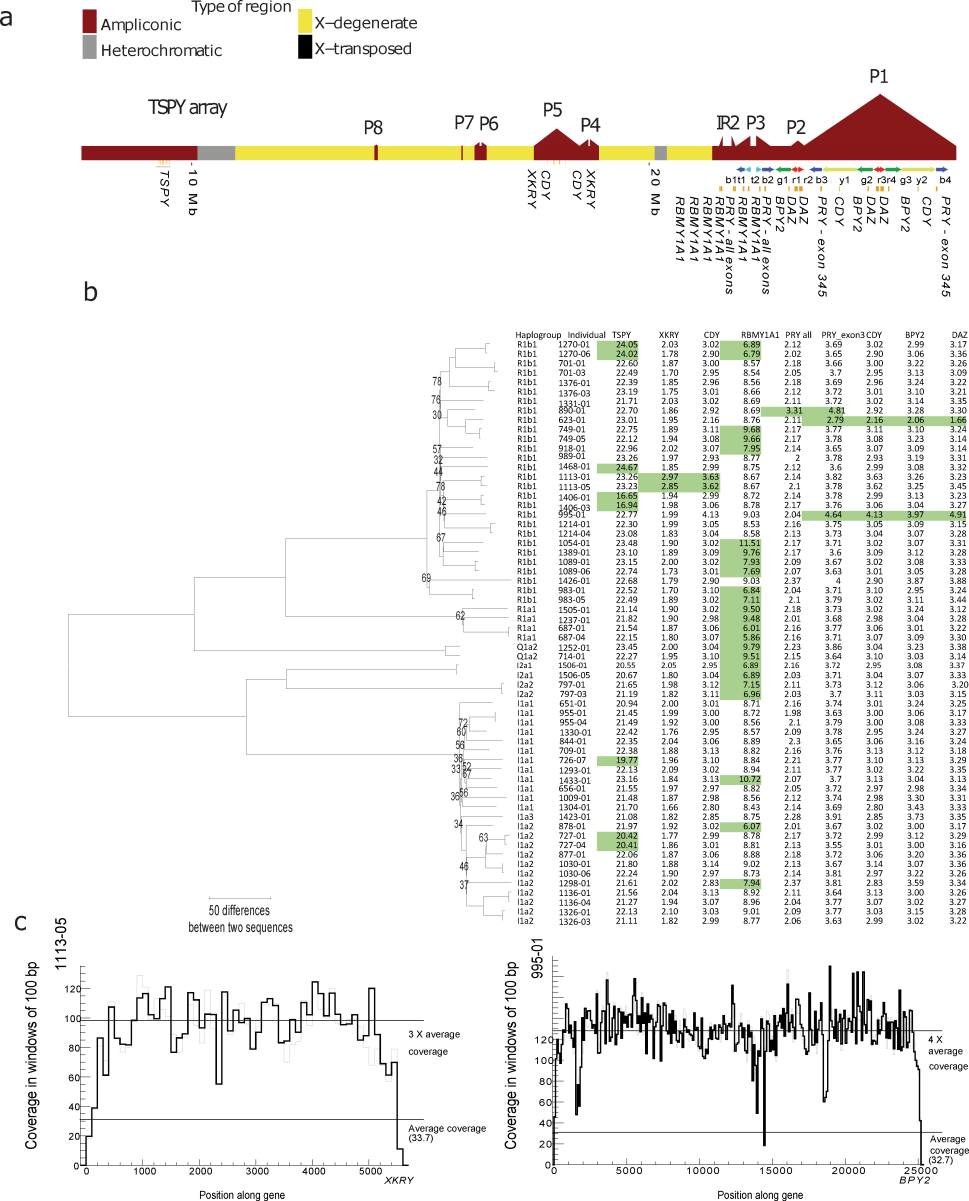
Gene duplications and deletions on the Y chromosome. **a)** A schematic of the Y chromosome is shown in the top with the palindromes marked and denoted P1-P8. The positions of the TSPY array and inverted repeat 2 (IR2) are also shown. Note that PRY is present in palindrome 3 with all exons and in palindrome 1 with exons 3, 4 and 5. **b)** To the left is a phylogenetic neighbor joining (NJ) tree showing the relationship between the different males; the numbers are bootstrap values. Bootstrap values above 90 are not shown. To the right is a table showing the haplogroup, name and copy number estimate for each individual. Genes where the copy number estimate differ from that of the reference sequence are colored green. For RBMY1A1 there are an unknown number of pseudogenes in the reference, but most individuals had 9 copies so this was chosen for the baseline. For clarity, only genes with copy number variants are shown. **c)** Two coverage profiles for two gene duplications, for individual 1113–05 in gene XKRY and one for individual 995–01 in BPY2.

To identify copy number variation, we mapped all sequence reads to one copy of each of the 24 distinct genes on the MSY except the X-transposed region. We normalized coverage to 1 Mb of X-degenerate region sequence and then estimated the copy number from the median read coverage along each gene compared to the 1 Mb region. For copy numbers in fathers and sons to be considered concordant we required normalized coverage to differ less than 0.5. We find that the concordance between the independently estimated copy numbers for father and son is 97.8% (399 matches of 408 pairs). All cases of non-concordance were found in *TSPY* and *VCY* with 5 and 4 dis-concordant father-son pairs respectively. There was never more than one copy number difference in these genes. Fig 4 summarizes the results based on the raw data found in supplementary file S4 Dataset.

The *RBMY1A1* gene changes copy number 15 times in the phylogeny. If we assume that there were 9 copies in the ancestor, we observe 8 independent deletions and 7 independent duplications.

Due to our inability to estimate *TSPY* copy numbers more precisely than to within one copy, we only count changes by more than 1 copy number from the rounded mean in the haplogroup, which was 22 for R1b, R1a and I1a, 23 for Q1a and 21 for I2a. Using this conservative approach, we find that *TSPY* changes copy number 5 times in our phylogeny. Many of the gene duplication and deletion events are probably linked due to their close proximity in the palindrome arms. It could also be possible that multiple independent gene duplications and gene losses have occurred, but given that there are already examples of duplication and deletions that include entire palindrome arms [12], it is more parsimonious that the events are linked.

We find an event that could be the known gr1/gr2 deletion where palindrome 2 and part of palindrome 1 is deleted which leads to a loss of copy of *BPY2* and *CDY*, three exons in *PRY* and two copies of *DAZ* in individual 623–01 [5]. We also found that this individual had half the coverage in the y1/y2 part of palindrome 1 compared to individuals in the same haplogroups, and very few pseudodiploid SNVs in the y1/y2 part of palindrome 1. All of this points towards a deletion of r1, r2, b3 and y1 for this individual.

We find higher copy numbers for all exons of *PRY* in individual 890–01. Since only exons 3, 4 and 5 are present in b3 and b4, the most likely explanation is a b1 or b2 duplication. We find a gr1/gr2 duplication event in 995–01 with one extra copy of *CDY*, *BPY2*, higher copy number for exons 3,4 and 5 in *PRY* and two copies of *DAZ*. Lastly, we find a novel duplication of part of palindrome 5 arm in a family 1113 leading to extra copies of *CDY* and *XRKY*. Only part of palindrome 5 is lacking pseudodiploid positions in this family, and this suggests that only a part of the arm has been duplicated. Most of these events are probably due to NAHR events, due to their presence in palindrome arms.

## Discussion

### High quality hybrid assemblies

We have shown that it is possible to assemble large parts of the human Y chromosome from ~40X short reads from multiple insert size libraries. Regions with high similarity to other chromosomes (X-transposed) do not have Y chromosome specific scaffolds, showing that the hybrid assembly approach cannot reliably distinguish regions that are more similar than 99%. This is also the reason why the palindrome arms are collapsed in the scaffolds.

The problem with collapsed palindromes was also a problem in the recent Gorilla Y chromosomes assembly [17]. To obtain fully assembled amplicons, methods like SHIMS [1] or very long reads from third generation sequencing methods are needed.

### SNV and indel calling

The availability of hybrid assemblies allows for indels larger than the read length to be identified, including large segments that have been deleted in the reference sequence. The 3326 bp insertion we find in haplogroup I, which shares 98% identity to the chimpanzee, has likely been in all Y chromosome haplogroups until recently, when it was deleted in haplogroups R and Q; thus it is missing from the reference genome and has therefore been missed by 1000Y or similar mapping-based initiatives. The heterochromatic regions are not usually included in analysis of the Y, but our high father-son concordance rate in this region suggest that it is should be included. Our results imply either that the point mutation rate in this region is up to an order of magnitude higher than the rest of the Y chromosome or that genetic variation has been introduced by non-homologous gene conversions from other chromosomes. The high similarity to other chromosomes in this region might suggest a rich history of transposition and a previous study found interchromosomal duplication events within 5 MB from the centromere on the Y chromosome and within 5 Mb of the centromere on chromosome 9 and 16 among others [30]. Since all of the variation is not shared between all individuals we would require multiple interchromosomal gene duplications to explain our data. We conclude that the observed 10-fold higher rate of polymorphism merits further studies to establish the mechanism.

### Understanding palindrome dynamics

We have, for the first time, inferred mutation and gene conversion events across all unique palindromes, which allowed us to investigate the gene conversions and mutation process in detail. We present evidence both of GC-biased gene conversion and conversion towards the ancestral state as suggested in previous much smaller studies [10,11]. The mutation process is biased towards AT, as in the rest of the genome, leading to a dynamic equilibrium where gene conversion is more likely to repair towards the ancestral state. We also find that in addition to this, gene conversion seems to favor the ancestral state even for mutations that do not change the GC content (27 gene conversions towards ancestral vs 17 gene conversions towards derived). Taken together these two effects slow down the evolution of palindrome sequence, explaining why we infer a lower mutation rate than in the X degenerate region (see also Helgason et al. 2015).

### Copy number of genes

Previous studies have shown that lower *TSPY* copy number and gr/gr deletions can increase the risk of spermatogenic failure [5,31]. However, all individuals in this study have no known diseases and all fathers produced sons or daughters, so the change in copy numbers does not seem to have a severe effect.

Our approach for identifying copy number of genes has limitations. The approach cannot distinguish between functional genes and pseudogenes as in the case with RBMY1A, which has 6 functional genes present in the reference sequence [1] and an unknown number of pseudogenes. This means that the copy number changes we observe could just as well be pseudogenes, which might not have an impact on an individual. To solve this problem, one would need mRNA expression data as well.

Moreover this method is not good at differentiating between copy numbers when they are very large, as in the case with *TSPY* where almost all our individuals have fewer copies than found in a previous study which used *Pme*I pulsed-field DNA blotting (between 26–28 copies in the haplogroups R,I and Q) [32]. This method also works best with uniform coverage across the gene but some genes contain repeats as in *DAZ* [33].

## Materials and methods

### Extraction and gap-closing of scaffolds

We constructed de novo assemblies with ALLPATHS-LG [34] using 7 different library insert sizes of 180, 500, 800, 2000, 5000, 10000 and 20000 bp see [18]. The scaffolds mapping to the Y chromosome when using LAST [22] were found.

In order for a scaffold to be kept it was required that the majority of the fragments were aligned to the Y chromosome and aligned with more than 1000 bp. This will remove fragments that align to the pseudo-autosomal region and X-transposed region and small scaffolds that align ambiguously across the genome. For closing the gaps within the scaffolds a program from SOAPdenovo2 called GapCloser [35] was used using reads from each of the 7 libraries. The first cycle started with the 180 bp insert library, the second with the 500 bp insert size and so on. The repeats in the scaffolds were masked using repeatmasker [36].

The scaffolds were sorted based on which order they mapped to the reference and reverse complemented if they mapped to the reverse strand.

### Concordance between father and sons

For each father son pair, the Y chromosome was broken down into regions (1^st^ ampliconic region. 2^nd^ ampliconic region. 1^st^ X-degenerate region. 2^nd^ X-degenerate region and so on) and scaffolds mapping to each region were extracted for each individual. The scaffolds were aligned using MAFFT [37] and regions identified by repeatmasker as repetitive were masked. Mismatches within 50 bp of alignment gaps were also masked. The concordance rate was reported for windows of 10 kb.

### Variation calling

Reads were mapped to the reference (GRCh38) using BWA-MEM version 0.7.5a [19] and refined by Stampy version 1.0.23 [38] both with standard parameters. GATK Haplotype caller version 3.2–2 was used for finding variants [20]. AsmVar was used for finding indels [21]. This was run using the gapclosed scaffolds.

### Merging and genotyping

The variants called with GATK and AsmVar was merged based on position, reference and alternative allele and genotyped using BayesTyper [23]. BayesTyper is a probabilistic framework for genotyping variants, that constructs a variant graph using all the input variants and the reference sequence. A library of k-mers of size 55 were constructed for all the reads coming from an individual. BayesTyper then checks how well a path through the variant graph is supported by the k-mers for an individual. The genotypes for all individuals are estimated jointly and only variants with a posterior probability greater than 0.9 with more than 3 k-mers supporting it were kept.

### Sanger validation of variants

We randomly selected 27 non-recurrent variants and 23 non-recurrent variants. For each variant, we picked one individual with the reference allele and one with the alternative. If the correct allele was present and we could sequence 50 bp up and downstream of the variant, we called the variant as validated.

### Construction of neighbor joining tree

The SNPs called using GATK were used for constructing the neighbor joining (NJ) tree. The SNPs were required to have a filter status of PASS, not be recurrent and they need to be in the X-degenerate region. The NJ tree was constructed using MEGA 6 [39] using the number of substitutions as the model and pairwise deletion as missing data treatment. It was run with 500 bootstrap replicates.

### Haplogroup assignment

Haplogroups were called with respect to a minimal list of SNPs [40] and the ISOGG database (International Society of Genetic Genealogy), Y-chromosome phylogeny, Y-DNA Haplogroup Tree 2016, Version: 11.239, Date: 2 September 2016, http://www.isogg.org/tree/.

If the haplogroup could not be identified the individual was assigned the haplogroup of the other individuals that it clusters with in the Neighbor joining tree.

### SNP calling in palindromes

The reads of each individual were mapped to all palindrome proximal arms using BWA-mem and filtered and sorted with Sambamba version 0.5.1 [41].

Reads were filtered away with Sambamba using the following criteria: "not (duplicate or secondary_alignment or unmapped) and mapping_quality > = 50 and cigar = ~ /100M/ and [NM] < 2". The reads cannot be duplicate, in secondary alignments or unmapped. Furthermore, the quality of the reads must be above 50, no insertions must be in the read and the number of mismatches in a read must be below 2. In addition the mate-paired reads above 10000 bp were not filtered with the ‘proper pair ‘ option.

SNPs were then called using platypus [42] with standard parameters other than “—maxReads 800000000—maxVariants 20”.

The coverage of each position was calculated using Samtools version 0.1.19 [43].

The SNPs were then filtered by requiring that each position had a depth of coverage between 50 and 250. Finally, the SNPs were phased using GATK with the parameters:

“—phaseQualityThresh 20.0—fix_misencoded_quality_scores -fixMisencodedQuals”.

### Estimation of event per position per generation

The number of events per position per generation (PPPG) for insertions, deletions, complex variants, STR mutations in the palindrome and gene conversions in the palindrome were calculated by dividing the number of events by the generations spanned by the tree and the length of the segment analyzed.

The total number of generations was calculated from the SNV mutation rate of 3.14 ∙ 10^−8^ PPPG [26] for 3126 SNVs called in 8.1 Mb of the X-degenerate region. The number of generations is estimated to be:

3126mutations3.14∙10−8mutationspositiongeneration∙8.1MB=12265generations

For mutations in palindromes there were 606 events and the length of the palindrome arms called was 2*1.4 Mb and the number of generations spanned by the tree was 12265 This means that the number of event PPPG was $\frac{606\;events}{12265\;generations∙2∙1.4\;Mb}=1.75∙10^{-8}$.

### Algorithm for calling gene conversions and mutation

In order to call gene conversion and mutation events in all palindromes we devised the following algorithm. First a phylogeny of all the samples must exist. We start with all the observed genotypes for all individuals in the tree. Then we do a bottom-up filling out of ancestral nodes based on their children. Next, we do a top-down assignment of events if the parent is different for one of the children. The method is illustrated in S3 Fig.

### Copy number variation

The coding sequence +/- 2 kb up- and downstream of 26 protein-coding genes on the Y chromosome was found using http://www.ncbi.nlm.nih.gov/ along with 1 Mb of X-degenerate region. The raw reads were mapped to these genes for each individual with BWA-mem and filtered using Sambamba with the parameters mentioned above.

CNVnator [44] was used to call duplications and deletions using a binsize of 100 bp and GC correction. CNVnator was used in the whole gene except for *DAZ* (only exon 28 was used–chrY: 23198797–23199094), *PRY* (exons 1 and 2, chrY: 22490396–22490484 and chrY: 2490585–22490672) and *TSPY* (FAM197Y2P –chrY: 9479053–9484654) was used.

### Ethics statement

This study has been approved of Den Nationale Videnskabsetiske komité (The Danish national committee on health research ethics) with approval number 1210920. All individuals participating have provided informed written consent.

### Data availability

Individual sequence data, alignment based assemblies for all 62 individuals in bam file format are available at the European Genome-phenome Archive (EGA), which is hosted by the EBI, under accession number EGAS00001002108. The variants used in this project has accession number EGAD00001003186. All other data, except the de novo assemblies, are within the paper and its Supporting Information files.

Due to patient confidentiality, access to the individual de novo assemblies can be accessed through agreement with the either Prof. Søren Brunak: soren.brunak@cpr.ku.dk, Prof. Mikkel Schierup: mheide@birc.au.dk or Prof. Karsten Kristiansen: kk@bio.ku.dk.

## Supporting information

S1 Dataset Hybrid assembly summary statistics. This spreadsheet contains 3 tabs; Scaffold statistics, Regions of the Y chromosome that lists the different reigons of the Y chromosome and Alignment to reference.
(XLSX)

S2 Dataset Structural variant calling. This spreadsheet contains 6 tabs; Variants that contains all called variants, Variants summary, Heterochrom BLAT that shows the similarity to other chromosomes of segments in the centromeric heterochromatic region, Validation results that show the Sanger validation for structural variants, Mutation rates, BLAST for large insertions.
(XLSX)

S3 Dataset Understanding palindrome dynamics. This spreadsheet contains two tabs; Length of palindrome arms used and Events.
(XLSX)

S4 Dataset Copy number variation. This spreadsheet contains two tabs; Copy number–not rounded and genes used.
(XLSX)

S5 Dataset Haplogroup identification. This spreadsheet contains one tab; Haplogroups.
(XLSX)

S1 Fig Assembly stats. (PDF)

S2 Fig Phylogeny construction. Neighbor joining vs. Maximum likelihood.
(PDF)

S3 Fig Method for determining mutations and gene conversions on the phylogeny. (PDF)

S4 Fig Evolutionary relationship between 62 samples in this study and the out-groups used. (PDF)

## References

[1] Skaletsky H, Kuroda-Kawaguchi T, Minx PJ, Cordum HS, Hillier L, et al (2003) The male-specific region of the human Y chromosome is a mosaic of discrete sequence classes. Nature 423: 825–837. doi: 10.1038/nature01722 12815422 10.1038/nature01722

[2] Bellott DW, Hughes JF, Skaletsky H, Brown LG, Pyntikova T, et al (2014) Mammalian Y chromosomes retain widely expressed dosage-sensitive regulators. Nature 508: 494–499. doi: 10.1038/nature13206 24759411 10.1038/nature13206 PMC4139287

[3] Cortez D, Marin R, Toledo-Flores D, Froidevaux L, Liechti A, et al (2014) Origins and functional evolution of Y chromosomes across mammals. Nature 508: 488–493. doi: 10.1038/nature13151 24759410 10.1038/nature13151

[4] Foresta C, Ferlin A, Moro E (2000) Deletion and expression analysis of AZFa genes on the human Y chromosome revealed a major role for DBY in male infertility. Hum Mol Genet 9: 1161–1169. 10767340 10.1093/hmg/9.8.1161

[5] Giachini C, Nuti F, Turner DJ, Laface I, Xue Y, et al (2009) TSPY1 copy number variation influences spermatogenesis and shows differences among Y lineages. The Journal of clinical endocrinology and metabolism 94: 4016–4022. 19773397 10.1210/jc.2009-1029 PMC3330747

[6] Ferlin A, Moro E, Garolla A, Foresta C (1999) Human male infertility and Y chromosome deletions: role of the AZF-candidate genes DAZ, RBM and DFFRY. Hum Reprod 14: 1710–1716. 10402373 10.1093/humrep/14.7.1710

[7] Nam K, Munch K, Hobolth A, Dutheil JY, Veeramah K, et al (2014) Strong selective sweeps associated with ampliconic regions in great ape X chromosomes. arXiv preprint arXiv:14025790.

[8] Mueller JL, Mahadevaiah SK, Park PJ, Warburton PE, Page DC, et al (2008) The mouse X chromosome is enriched for multicopy testis genes showing postmeiotic expression. Nat Genet 40: 794–799. doi: 10.1038/ng.126 18454149 10.1038/ng.126 PMC2740655

[9] Soh YQ, Alfoldi J, Pyntikova T, Brown LG, Graves T, et al (2014) Sequencing the mouse Y chromosome reveals convergent gene acquisition and amplification on both sex chromosomes. Cell 159: 800–813. doi: 10.1016/j.cell.2014.09.052 25417157 10.1016/j.cell.2014.09.052 PMC4260969

[10] Hallast P, Balaresque P, Bowden GR, Ballereau S, Jobling MA (2013) Recombination Dynamics of a Human Y-Chromosomal Palindrome: Rapid GC-Biased Gene Conversion, Multi-kilobase Conversion Tracts, and Rare Inversions. PLoS Genetics 9. 10.1371/journal.pgen.1003666 PMC3723533 23935520

[11] Rozen S, Skaletsky H, Marszalek JD, Minx PJ, Cordum HS, et al (2003) Abundant gene conversion between arms of palindromes in human and ape Y chromosomes. Nature 423: 873–876. doi: 10.1038/nature01723 12815433 10.1038/nature01723

[12] Jobling MA (2008) Copy number variation on the human Y chromosome. Cytogenet Genome Res 123: 253–262. doi: 10.1159/000184715 19287162 10.1159/000184715

[13] Poznik GD, Xue Y, Mendez FL, Willems TF, Massaia A, et al (2016) Punctuated bursts in human male demography inferred from 1,244 worldwide Y-chromosome sequences. Nat Genet 48: 593–599. doi: 10.1038/ng.3559 27111036 10.1038/ng.3559 PMC4884158

[14] Johansson MM, Van Geystelen A, Larmuseau MH, Djurovic S, Andreassen OA, et al (2015) Microarray Analysis of Copy Number Variants on the Human Y Chromosome Reveals Novel and Frequent Duplications Overrepresented in Specific Haplogroups. PLoS One 10: e0137223 doi: 10.1371/journal.pone.0137223 26322892 10.1371/journal.pone.0137223 PMC4554990

[15] Wei W, Fitzgerald TW, Ayub Q, Massaia A, Smith BH, et al (2015) Copy number variation in the human Y chromosome in the UK population. Hum Genet 134: 789–800. doi: 10.1007/s00439-015-1562-5 25957587 10.1007/s00439-015-1562-5 PMC4460274

[16] Hughes JF, Skaletsky H, Pyntikova T, Graves TA, van Daalen SK, et al (2010) Chimpanzee and human Y chromosomes are remarkably divergent in structure and gene content. Nature 463: 536–539. 20072128 10.1038/nature08700 PMC3653425

[17] Tomaszkiewicz M, Rangavittal S, Cechova M, Campos Sanchez R, Fescemyer HW, et al (2016) A time- and cost-effective strategy to sequence mammalian Y Chromosomes: an application to the de novo assembly of gorilla Y. Genome Res. 10.1101/gr.199448.115 PMC4817776 26934921

[18] Maretty L, Jensen JM, Petersen B, Sibbesen JA, Liu S, et al (2017) Sequencing and de novo assembly of 150 genomes from Denmark as a population reference. Nature 548: 87–91. doi: 10.1038/nature23264 28746312 10.1038/nature23264

[19] Li H, Durbin R (2009) Fast and accurate short read alignment with Burrows-Wheeler transform. Bioinformatics (Oxford, England) 25: 1754–1760. 10.1093/bioinformatics/btp324 PMC2705234 19451168

[20] McKenna A, Hanna M, Banks E, Sivachenko A, Cibulskis K, et al (2010) The Genome Analysis Toolkit: a MapReduce framework for analyzing next-generation DNA sequencing data. Genome Res 20: 1297–1303. doi: 10.1101/gr.107524.110 20644199 10.1101/gr.107524.110 PMC2928508

[21] Liu S, Huang S, Rao J, Ye W, Genome Denmark Consortium II, et al (2015) Discovery, genotyping and characterization of structural variation and novel sequence at single nucleotide resolution from de novo genome assemblies on a population scale. Gigascience 4: 64 doi: 10.1186/s13742-015-0103-4 26705468 10.1186/s13742-015-0103-4 PMC4690232

[22] Frith MC, Hamada M, Horton P (2010) Parameters for accurate genome alignment. BMC bioinformatics. 10.1186/1471-2105-11-80 PMC2829014 20144198

[23] Sibbesen JA, Maretty L, Krogh A (2017) BayesTyper [Internet]. [place unknown]: Github.

[24] Genomes Project C, Auton A, Brooks LD, Durbin RM, Garrison EP, et al (2015) A global reference for human genetic variation. Nature 526: 68–74. doi: 10.1038/nature15393 26432245 10.1038/nature15393 PMC4750478

[25] Altschul SF, Gish W, Miller W, Myers EW, Lipman DJ (1990) Basic local alignment search tool. J Mol Biol 215: 403–410. 2231712 10.1016/S0022-2836(05)80360-2

[26] Helgason A, Einarsson AW, Guðmundsdóttir VB, Sigurðsson Á, Gunnarsdóttir ED, et al (2015) The Y-chromosome point mutation rate in humans. Nature genetics. 10.1038/ng.3171 25807285

[27] Karmin M, Saag L, Vicente M, Wilson Sayres MA, Jarve M, et al (2015) A recent bottleneck of Y chromosome diversity coincides with a global change in culture. Genome Res 25: 459–466. doi: 10.1101/gr.186684.114 25770088 10.1101/gr.186684.114 PMC4381518

[28] Kent WJ (2002) BLAT—the BLAST-like alignment tool. Genome Res 12: 656–664. doi: 10.1101/gr.229202 11932250 10.1101/gr.229202 PMC187518

[29] Mallick S, Li H, Lipson M, Mathieson I, Gymrek M, et al (2016) The Simons Genome Diversity Project: 300 genomes from 142 diverse populations. Nature 538: 201–206. doi: 10.1038/nature18964 27654912 10.1038/nature18964 PMC5161557

[30] She XW, Horvath JE, Jiang ZS, Liu G, Furey TS, et al (2004) The structure and evolution of centromeric transition regions within the human genome. Nature 430: 857–864. doi: 10.1038/nature02806 15318213 10.1038/nature02806

[31] Repping S, Skaletsky H, Brown L, van Daalen SK, Korver CM, et al (2003) Polymorphism for a 1.6-Mb deletion of the human Y chromosome persists through balance between recurrent mutation and haploid selection. Nat Genet 35: 247–251. doi: 10.1038/ng1250 14528305 10.1038/ng1250

[32] Repping S, van Daalen SK, Brown LG, Korver CM, Lange J, et al (2006) High mutation rates have driven extensive structural polymorphism among human Y chromosomes. Nat Genet 38: 463–467. doi: 10.1038/ng1754 16501575 10.1038/ng1754

[33] Saxena R, de Vries JW, Repping S, Alagappan RK, Skaletsky H, et al (2000) Four DAZ genes in two clusters found in the AZFc region of the human Y chromosome. Genomics 67: 256–267. doi: 10.1006/geno.2000.6260 10936047 10.1006/geno.2000.6260

[34] Gnerre S, Maccallum I, Przybylski D, Ribeiro FJ, Burton JN, et al (2011) High-quality draft assemblies of mammalian genomes from massively parallel sequence data. Proc Natl Acad Sci U S A 108: 1513–1518. doi: 10.1073/pnas.1017351108 21187386 10.1073/pnas.1017351108 PMC3029755

[35] Luo R, Liu B, Xie Y, Li Z, Huang W, et al (2012) SOAPdenovo2: an empirically improved memory-efficient short-read de novo assembler. Gigascience 1: 18 doi: 10.1186/2047-217X-1-18 23587118 10.1186/2047-217X-1-18 PMC3626529

[36] Smit AFA, Hubley R, Green P (2013) RepeatMasker Open 4.0. RepeatMasker Open 40.

[37] Katoh K, Standley DM (2013) MAFFT multiple sequence alignment software version 7: improvements in performance and usability. Molecular biology and evolution. 10.1093/molbev/mst010 PMC3603318 23329690

[38] Lunter G, Goodson M (2011) Stampy: a statistical algorithm for sensitive and fast mapping of Illumina sequence reads. Genome Res 21: 936–939. 20980556 10.1101/gr.111120.110 PMC3106326

[39] Tamura K, Stecher G, Peterson D, Filipski A, Kumar S (2013) MEGA6: Molecular Evolutionary Genetics Analysis version 6.0. Molecular biology and evolution 30: 2725–2729. doi: 10.1093/molbev/mst197 24132122 10.1093/molbev/mst197 PMC3840312

[40] Oven M, Geystelen A, Kayser M, Decorte R, Larmuseau MHD (2014) Seeing the Wood for the Trees: A Minimal Reference Phylogeny for the Human Y Chromosome. Human Mutation 35: 187–191. doi: 10.1002/humu.22468 24166809 10.1002/humu.22468

[41] Tarasov A, Vilella AJ, Cuppen E, Nijman IJ (2015) Sambamba: fast processing of NGS alignment formats. 10.1093/bioinformatics/btv098 PMC4765878 25697820

[42] Rimmer A, Phan H, Mathieson I, Iqbal Z, Twigg SRF (2014) Integrating mapping-, assembly-and haplotype-based approaches for calling variants in clinical sequencing applications. Nature. 10.1038/ng.3036 PMC4753679 25017105

[43] Li H, Handsaker B, Wysoker A, Fennell T, Ruan J, et al (2009) The Sequence Alignment/Map format and SAMtools. Bioinformatics 25: 2078–2079. doi: 10.1093/bioinformatics/btp352 19505943 10.1093/bioinformatics/btp352 PMC2723002

[44] Abyzov A, Urban AE, Snyder M, Gerstein M (2011) CNVnator: an approach to discover, genotype, and characterize typical and atypical CNVs from family and population genome sequencing. Genome Res 21: 974–984. doi: 10.1101/gr.114876.110 21324876 10.1101/gr.114876.110 PMC3106330

