# Supplementary figures and images for: Analysis of 62 hybrid assembled human Y chromosomes exposes rapid structural changes and high rates of gene conversion
Source: PLoS Genet. 2017 Aug 28;13(8):e1006834. doi: 10.1371/journal.pgen.1006834 (PMC5591018; doi:10.1371/journal.pgen.1006834)

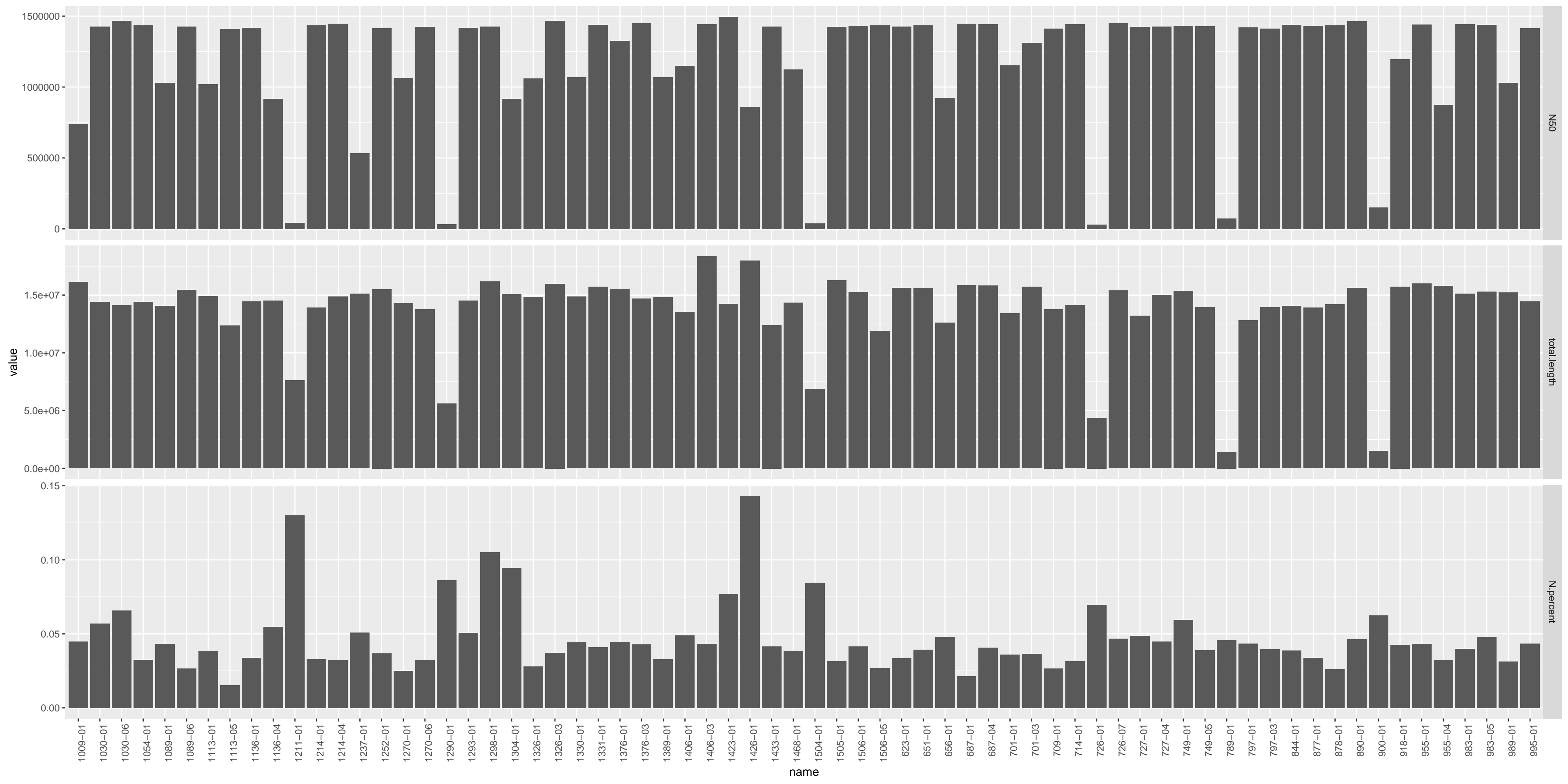

Supplement: S1 Fig — (PDF) [file pgen.1006834.s006.pdf]

# Evolutionary relationship between samples in this study and the outgroups used

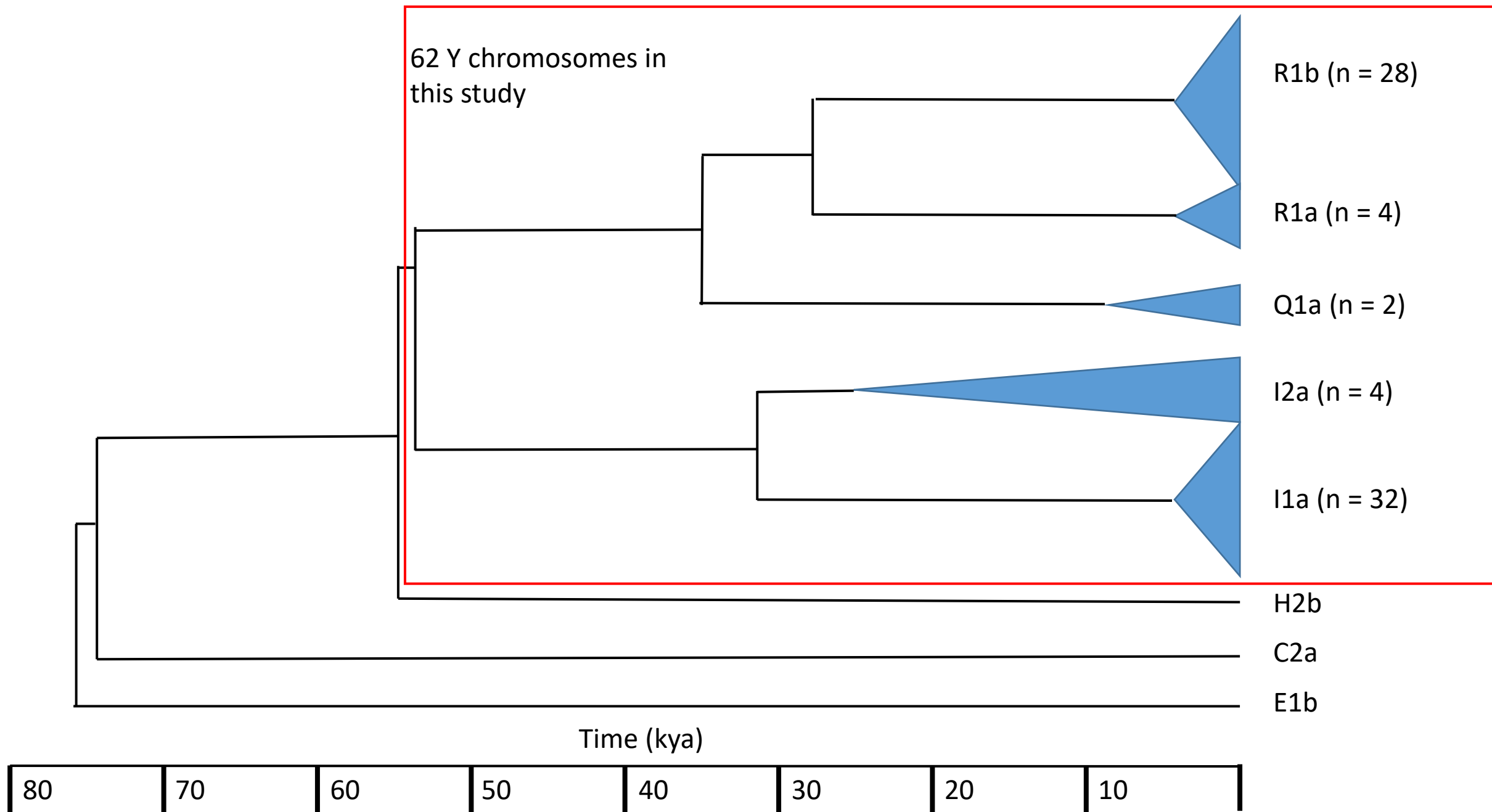

Supplement: S4 Fig — (PDF) [file pgen.1006834.s009.pdf]
