# Supplementary material for: Analysis of 62 hybrid assembled human Y chromosomes exposes rapid structural changes and high rates of gene conversion
Source: PLoS Genet. 2017 Aug 28;13(8):e1006834. doi: 10.1371/journal.pgen.1006834 (PMC5591018; doi:10.1371/journal.pgen.1006834)

ML tree misplaces individual 709-01, which carries the derived state for SNP P109, and therefore belongs to haplogroup I1a1b1

NJ tree – 500 bootstrap

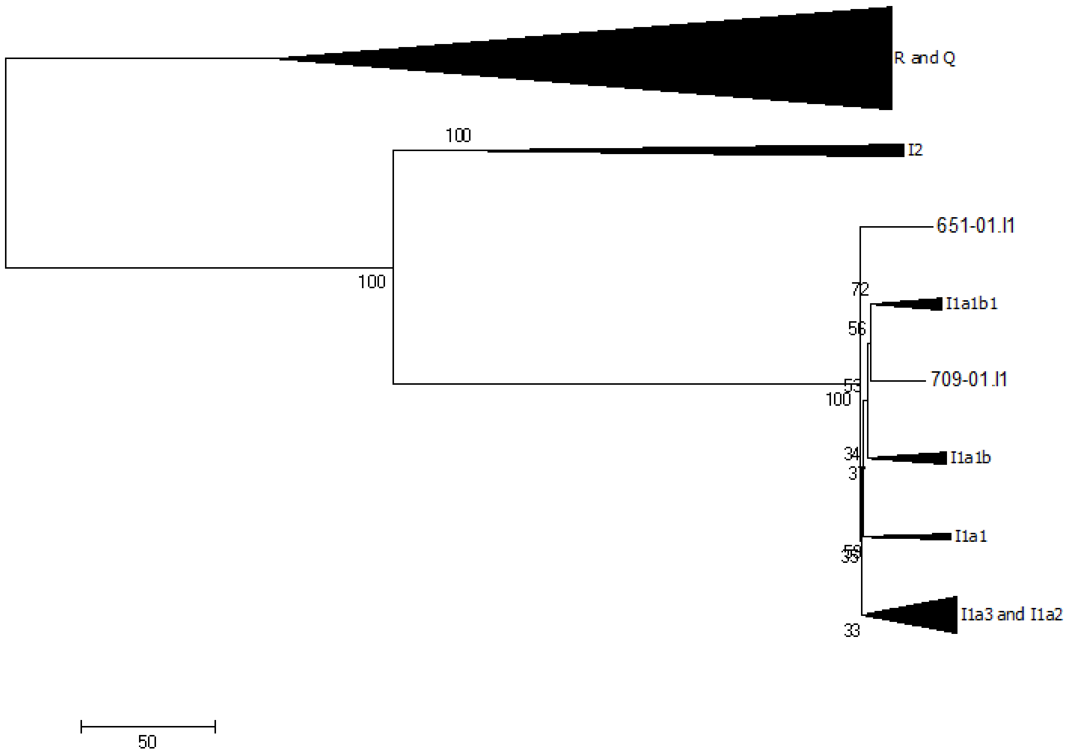

ML tree – 500 bootstrap

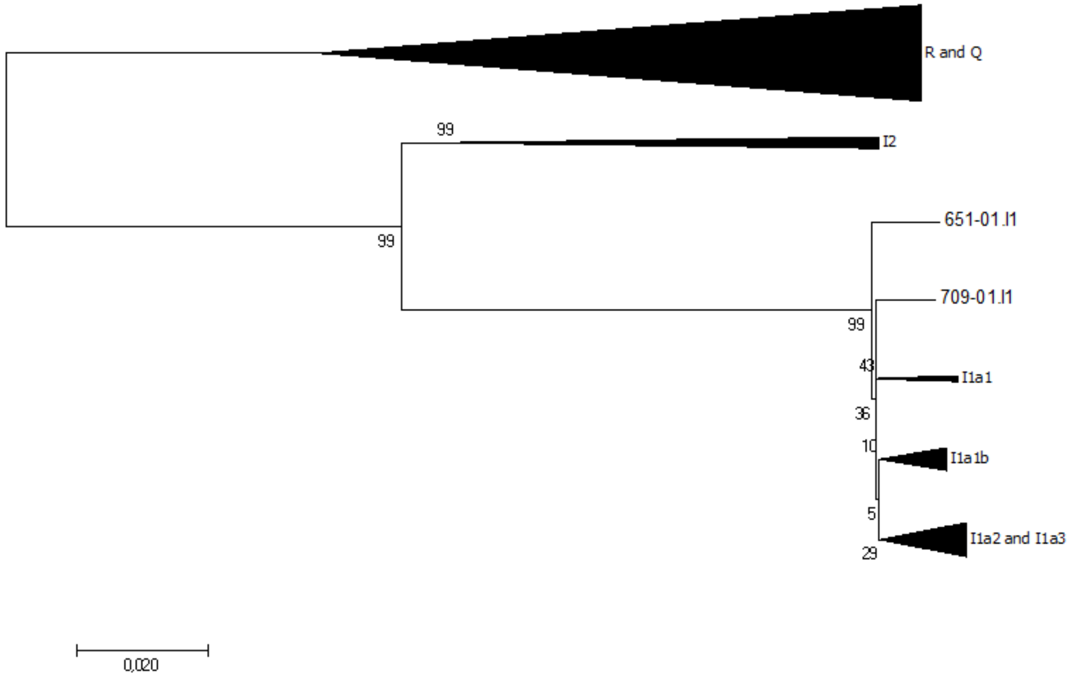

Supplement: S2 Fig — Neighbor joining vs. Maximum likelihood. (PDF) [file pgen.1006834.s007.pdf]
