# Supplementary material for: Analysis of 62 hybrid assembled human Y chromosomes exposes rapid structural changes and high rates of gene conversion
Source: PLoS Genet. 2017 Aug 28;13(8):e1006834. doi: 10.1371/journal.pgen.1006834 (PMC5591018; doi:10.1371/journal.pgen.1006834)
Supplement: S3 Fig — (PDF) [file pgen.1006834.s008.pdf]

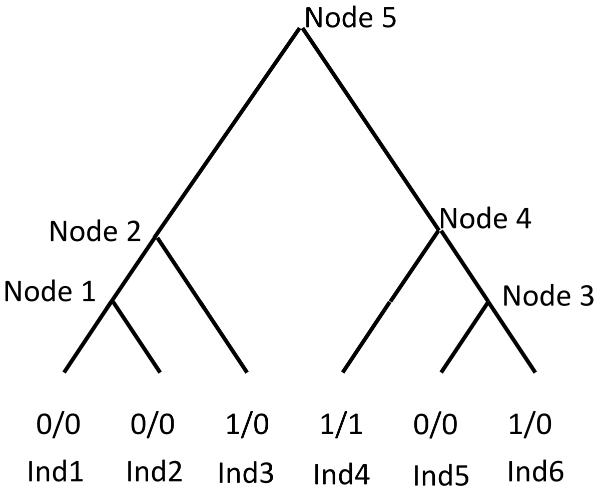

A phylogeny of the individuals present must exist. All the genotypes of each individual must be known

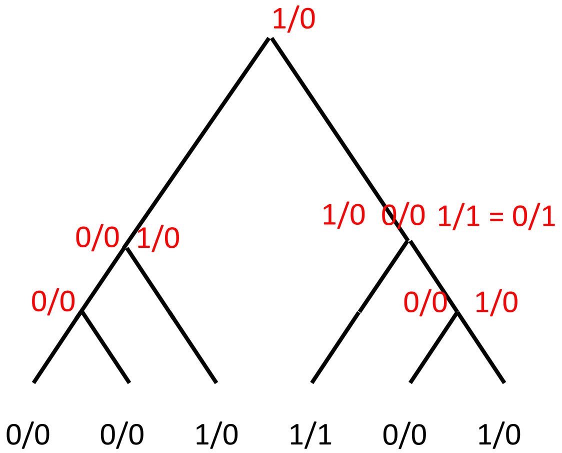

Call the ancestral genotype in nodes 1 to 5.

Node 1 – both children are 0/0 so this will be too
Node 2 – the child nodes are Node 1 and Ind3 who are 0/0 and 1/0. We don't know which is the right one so we keep both
Node 3 – Children are 0/0 and 0/1 so it is the same situation as Node 2.
Node 4 – Children are (0/0 and 1/0) from Node 3 and 1/1 from Ind4. To avoid multiple mutations we make this state 0/1. This will explain the data with two gene conversions.
Node 5 – The children are Node 2 with (0/0 and 0/1) and Node 4 with 0/1. The most parsimonious would be to make this 0/1.

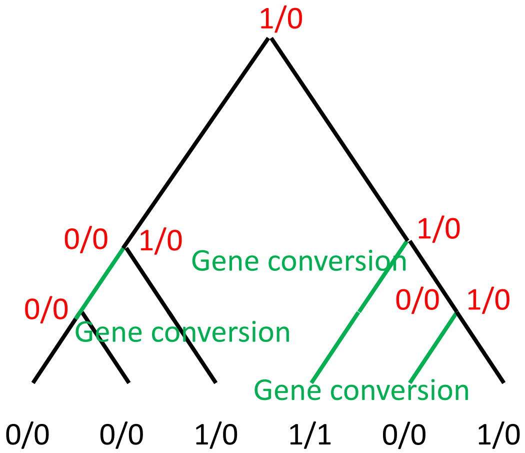

Infere events

Here we start from the top of the tree.

Node 5 - has two children Node 4 and Node 2. Since Node 4 is also 1/0 and Node 3 could either be 0/0 and 1/0, no events are infere here.
Node 4 – has two children Ind4 and Node3. Since Node 3 could be either 1/0 or 0/0 no event is inferred here. For Ind4 this genotype 1/1 is different from 0/1 and can be explained by a gene conversion.
Node 3 – The children are Ind5 and Ind6. The only difference is with ind5 which can be explained by a gene conversion.
Node 4 – The difference is with the child Node 1 which is 0/0 which can be explained with a gene conversion.
Node 5 – no events

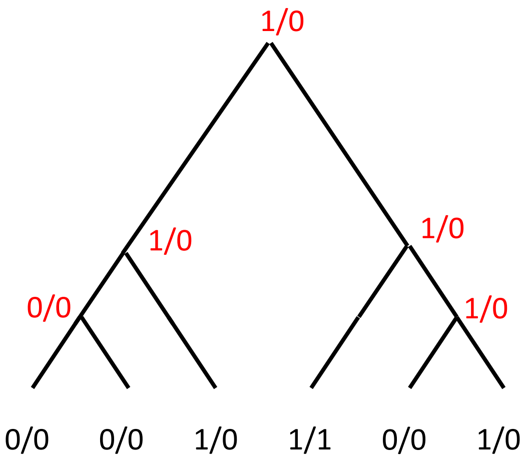

Summarize findings

Write up the events found.
3 Gene conversions

1 between Node 1 and Node 2
1 between Ind4 and Node 4
1 between Ind5 and Node 3
